# Treatment of Spontaneous Aneurism by Rest and Absolute Diet

**Published:** 1853-04

**Authors:** 


*Treatment of Spontaneous Aneurism by Rest and Absolute Diet.* From the Report of Prof. Gross to the Kentucky Medical Society.—Prof. Bush, of Lexington, has communicated to me the particulars of a case of spontaneous aneurism of the abdominal aorta, in which marked relief of the symptoms seems to have followed the observance of a most rigorous diet. The patient, Mrs. Anderson, a midwife, aged sixty, resides in Madison county, Kentucky, and had led a very exposed and laborious life for many years. When Dr. Bush first saw her, about three months ago, the tumor, situated just below the stomach, was about the size of a pullet's egg, and pulsated most violently, emitting all the usual aneurismal sounds. The heart was involved in the trouble, laboring and irregular in its actions. Believing that the case would be fatal, Dr. Bush merely advised quietude and absolute diet, barely enough of the lightest and least stimulating articles to support life. For a short time she grew worse, and her physician Dr. Evans, informed Dr. Bush that a *post-mortem* exanimation could be obtained when she died. Not long afterwards he learned that, under a rigid adherence to the treatment, the woman was rapidly improving, in fact, getting well, with a decided subsidence of all the local symptoms. "This," adds Dr. Bush, "is the whole of my experience in spontaneous aneurism, excepting one or two cases which I have seen in the hands of Dr. Dudley, treated in the same manner, but not of Kentucky."

The starving plan of treatment, first recommended by Valsalva, and so happily employed by Dr. Bush in the above instance, is worthy of the serious consideration of the surgeon in all cases of spontaneous aneurism, inaccessible to the ligature. When properly carried out, it may not only retard the fatal progress of the disease, but occasionally even effect a cure, especially in the milder and more recent forms of the affection. Valsalva's plan, as is well known, was to subject his patients to the most perfect rest in the horizontal posture, and to diminish the quantity of food gradually, till only half a pint of soup was allowed in the morning, and a quarter of a pint in the evening, with a very small quantity of water, medicated with osteocolla, or mucilage of quinces.* This treatment was aided, particularly in robust subjects, by the repeated abstraction of blood. Professor Dudley asserts that he has cured some cases simply by restricted diet, without the use of the lancet; and a recent foreign journal mentions several instances of a similar kind relieved by Dr. Bellingham, of Dublin.

* Cooper's Surgical Dictionary, art. Aneurism.

